# Risk factors, clinical features, and outcomes of premature acute myocardial infarction

**DOI:** 10.3389/fcvm.2022.1012095

**Published:** 2022-11-30

**Authors:** Qi Liu, Rui-Juan Shi, Yi-Man Zhang, Yi-Heng Cheng, Bo-Sen Yang, Yi-Ke Zhang, Bao-Tao Huang, Mao Chen

**Affiliations:** Department of Cardiology, West China Hospital, Sichuan University, Chengdu, China

**Keywords:** CAD, acute myocardial infarction, risk factors, prognosis, premature

## Abstract

**Aims:**

To investigate the risk factors, clinical features, and prognostic factors of patients with premature acute myocardial infarction (AMI).

**Materials and methods:**

A retrospective cohort study of patients with AMI included in data from the West China Hospital of Sichuan University from 2011 to 2019 was divided into premature AMI (aged < 55 years in men and < 65 years in women) and non-premature AMI. Patients’ demographics, laboratory tests, Electrocardiography (ECG), cardiac ultrasound, and coronary angiography reports were collected. All-cause death after incident premature MI was enumerated as the primary endpoint.

**Results:**

Among all 8,942 AMI cases, 2,513 were premature AMI (79.8% men). Compared to the non-premature AMI group, risk factors such as smoking, dyslipidemia, overweight, obesity, and a family history of coronary heart disease (CHD) were more prevalent in the premature AMI group. The cumulative survival rate of patients in the premature AMI group was significantly better than the non-premature AMI group during a mean follow-up of 4.6 years (HR = 0.27, 95% CI 0.22–0.32, *p* < 0.001). Low left ventricular ejection fraction (LVEF) (Adjusted HR 3.00, 95% CI 1.85–4.88, *P* < 0.001), peak N-terminal pro-B-type natriuretic peptide (NT-proBNP) level (Adjusted HR 1.34, 95% CI 1.18–1.52, *P* < 0.001) and the occurrence of in-hospital major adverse cardiovascular and cerebrovascular events (MACCEs) (Adjusted HR 2.36, 95% CI 1.45–3.85, *P* = 0.001) were predictors of poor prognosis in premature AMI patients.

**Conclusion:**

AMI in young patients is associated with unhealthy lifestyles such as smoking, dyslipidemia, and obesity. Low LVEF, elevated NT-proBNP peak level, and the occurrence of in-hospital MACCEs were predictors of poor prognosis in premature AMI patients.

## Introduction

Coronary heart disease (CHD) has always been the number one killer threatening human health. According to the top ten causes of death globally, the World health organization (WHO) released in 2019, ischemic heart disease still occupies the top 16% of the total death (1). Zhou’s research conducted a systematic analysis using the data from the Global Burden of Disease Study 2017 (GBD 2017), which found that ischemic heart disease was the second leading cause of death in China behind stroke (2).

Traditional risk factors in the development of CHD include smoking, a history of diabetes, hypertension, and dyslipidemia. As one of the independent risk factors, age tends to show a gradual increase in the incidence of CHD as age increases. Based on previous research, young patients with acute myocardial infarction (AMI) account for approximately 2–6% of all (3). However, data showed that the proportion of young people hospitalized for AMI is stable or increasing in many countries (4, 5). At the same time, the age of the first occurrence of AMI is becoming younger (4).

Young patients with myocardial infarction differ from older patients in etiology, risk factors, clinical features, treatment, and prognosis. Given that young individuals have a longer life expectancy and higher demands on the quality of life, conducting in-depth studies on this group’s clinical and prognostic indicators is essential. A few studies give us some clue on the prevalence, characteristics, and prognosis of premature AMI in Europe and the Americas area (6–12). The present study provides evidence on risk factors, clinical features, and in-hospital and mid-to-long-term prognosis of premature AMI in China.

## Materials and methods

### Study design and population

This study selected patients (*n* = 8,972) with acute myocardial infarction as a primary diagnosis who were admitted to the Department of Cardiology in West China Hospital of Sichuan University from January 1, 2011 to June 30, 2019. Screening according to the following inclusion and exclusion criteria, 8,942 patients were enrolled. According to previous studies (13–15), premature AMI was defined as the first occurrence of AMI aged < 55 years in men and < 65 years in women. Based on this definition, patients were divided into premature AMI (*n* = 2,513) and non-premature AMI (*n* = 6,429) groups. 415 patients who died in the hospital were not included in the outcome analysis. Detailed patient flow can be seen in Scheme 1.

Inclusion criteria: patients with a diagnosis consistent with the Fourth Universal Definition of Myocardial Infarction (16).

Exclusion criteria: incomplete data; < 18 years old; myocardial injury due to the following causes: interventional procedures, cardiac and non-cardiac surgery, heart failure, trauma, and infectious shock.

The study was approved by the Ethics Committee of the West China Hospital of Sichuan University (2012-243).

### Study endpoints and data

The primary endpoint was all-cause mortality at a follow-up that begins with patient discharge and ends on 2021-01-01. Loss of follow-up was defined as the inability to obtain information about the patient’s survival before the cut-off time. The second endpoints included all-cause death, cardiovascular death, and major adverse cardiovascular and cerebrovascular events (including cardiogenic shock, malignant arrhythmia, post-MI mechanical complications, non-fatal stroke, and non-fatal MI) in the hospital.

Parameters collected from the patients’ medical records at the time of hospitalization included: age at the time of AMI, gender, height, weight, smoking history; history of hypertension, diabetes, dyslipidemia, chronic kidney disease (CKD), peripheral arterial disease (PAD) and stroke, and family history of CHD. Data obtained during hospitalization included: hemoglobin, leukocyte count, platelet count, N-terminal pro-B-type natriuretic peptide (NT-proBNP) peak, cardiac troponin T(cTnT) peak, blood glucose level, lipid levels [cholesterol, triglycerides, high-density lipoprotein cholesterol (HDL-C), low-density lipoprotein cholesterol (LDL-C)], serum potassium level, electrocardiogram results and echocardiography results. For those who have had coronary angiography, collect the angiogram results.

### Statistical analysis

Data analysis was performed using IBM SPSS statistics 26.0. The Shapiro-Wilk test was used to assess the normality of distribution. Continuous variables are present as means (standard deviation) or median (interquartile range). *T*-test or Mann-Whitney *U* test (two groups of independent samples) were used to compare the continuous variables. Categorical variables were expressed as numbers or percentages and compared by Pearson’s Chi-square test or Fisher’s exact test. Missing data were interpolated by expectation maximization. Survival curves for time-to-event variables were demonstrated by Kaplan-Meier estimates and compared by a log-rank test. Multivariable analyses with the Cox proportional-hazards model were used to estimate the simultaneous effects of prognostic factors on survival. Non-linear trends and the changes in a given trend were analyzed using the Joinpoint Regression Program (version 4.9.0.0, March 2021; Statistical Methodology and Applications Branch, Surveillance Research Program, National Cancer Institute, Bethesda, MA, USA). All *p* values were two-tailed, and *p* < 0.05 for analyses was considered statistically significant. Notedly, all the comparable results were expressed as Premature AMI vs. Non-premature AMI or Men vs. Women.

## Results

**FIGURE 1:**
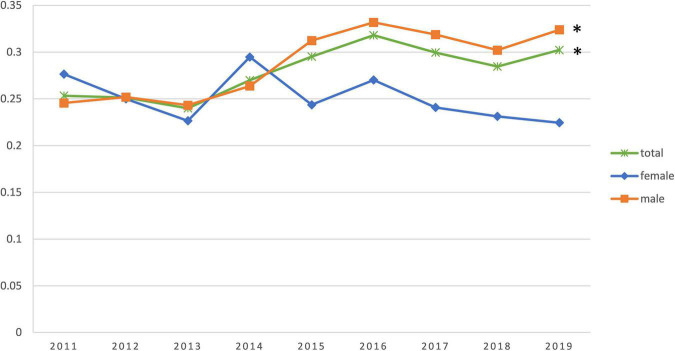
Trends in the percentage change of young patients in the annual acute myocardial infarction (AMI) incidence. *The difference is statistically significant.

The study population consisted of 8,942 AMI patients (77.2% men) with a mean age of 64.3 (± 12.97) years [70.7 (± 10.82) years in women and 62.5 (± 12.96) years in men]. Based on the grouping described earlier, the mean age was 48.4 (± 7.41) years in the premature AMI group and 70.6 (± 8.65) years in the non-premature AMI group. The premature AMI group was 79.8% male compared to 76.2% male in the non-premature AMI group (*P* < 0.001). For the composition ratio of the premature AMI group to the total number of admissions for AMI per year, we performed a Joinpoint regression. We observed an increase in the composition ratio of premature AMI from 2011 to 2019 (APC 2.85%, 95% CI 0.9%∼4.9%, *P* = 0.011). This trend is statistically significant in men (APC 4.10%, 95% CI 1.90%∼6.40%, *P* = 0.003), but not in women (APC −1.70%, 95% CI −4.30%∼1.00%, *P* = 0.176) (Figure 1, Supplementary Figure 1, and Supplementary Table 1).

### Risk factors and pathogeny

In the overall population, dyslipidemia (62.3%), hypertension (47.3%), smoking (46.4%), overweight (27.7%), and diabetes mellitus (21.8%) were the most prevalent risk factors. Compared to the non-premature AMI group, risk factors such as smoking (65.7 vs. 38.9%, *P* < 0.001), dyslipidemia (75.5 vs. 57.1%, *P* < 0.001), overweight (60.7 vs. 14.8%, *P* < 0.001), obesity (4.1 vs. 1.6%, *P* < 0.001) and a family history of CHD (7.0 vs. 2.8%, *P* < 0.001) were more prevalent in the premature AMI group. Meanwhile, hypertension, diabetes, CKD, and stroke were more common in the non-premature AMI group (Table 1). As shown in Supplementary Table 2, 97.8% of patients had at least one risk factor in the premature AMI group, compared with 92.1% in the non-premature AMI group (*P* < 0.001).

**TABLE 1:** Baseline demographic, risk factors, and clinical features by age.

	Premature AMI (*n* = 2,513)	Non-premature AMI (*n* = 6,429)	*P*-value
Age, years	48.4 (± 7.41)	70.6 (± 8.65)	< 0.001
Men, n (%)	2005 (79.8)	4901 (76.2)	< 0.001
**Risk factors AND pathogeny**			
Current smoker, n (%)	1651 (65.7)	2503 (38.9)	< 0.001
Hypertension, n (%)	811 (32.3)	3423 (53.2)	< 0.001
Diabetes, n (%)	407 (16.2)	1544 (24.0)	< 0.001
Dyslipidemia, n (%)	1897 (75.5)	3673 (57.1)	< 0.001
Overweight, n (%)	1526 (60.7)	949 (14.8)	< 0.001
Obesity, n (%)	102 (4.1)	100 (1.6)	< 0.001
Family history of CHD, n (%)	177 (7.0)	181 (2.8)	< 0.001
History of stroke, n (%)	49 (2.0)	384 (6.0)	< 0.001
History of CKD, n (%)	50 (2.0)	252 (3.9)	< 0.001
History of PAD, n (%)	16 (0.6)	48 (0.7)	0.576
ST-segment elevation, n (%)	1653 (65.8)	3455 (53.7)	< 0.001
**Clinical features**			
Typical chest pain, n (%)	1296 (51.6)	2909 (45.2)	< 0.001
Anterior, n (%)	914 (36.4)	1897 (29.5)	< 0.001
Inferior, n (%)	752 (29.9)	1595 (24.8)	< 0.001
Lateral, n(%)	65 (2.6)	98 (1.5)	0.001
Killip class II–IV, n (%)	759 (30.2)	3510 (54.6)	< 0.001
**Vitals on admission**			
HR, bpm	79 (70, 92)	78 (68, 90)	0.027
MAP, mmHg	91 (81, 103)	90 (80, 101)	< 0.001
LVEF < 50%(*n* = 7525)	667 (31.4)	2137 (39.5)	< 0.001
**Laboratory tests**			
WBC, × 10^9^/L	10.2 (7.7, 13.0)	8.9 (6.9, 11.7)	< 0.001
Hb, g/L	145 (131, 155)	130 (116, 143)	< 0.001
PLT, × 10^9^/L	187 (145, 236)	162 (125, 205)	< 0.001
GLU, mmol/L	7.16 (5.86, 9.54)	7.51 (6.06, 10.0)	0.003
Cr, μmol/L	77 (66, 90)	86 (72, 109)	< 0.001
UA, μmol/L	370 (301, 433)	357 (292, 439)	0.360
TG, mmol/L	1.66 (1.15, 2.55)	1.25 (0.92, 1.76)	< 0.001
TC, mmol/L	4.49 (3.69, 5.33)	4.07 (3.42, 4.83)	< 0.001
LDL-C, mmol/L	2.75 (2.07, 3.44)	2.40 (1.85, 3.06)	< 0.001
HDL-C, mmol/L	1.03 (0.84, 1.24)	1.13 (0.92, 1.37)	< 0.001
K^+^, mmol/L	3.80 (3.52, 4.11)	3.91 (3.60, 4.26)	< 0.001
cTnT peak, ng/L	1607.5 (377.6, 3767.0)	1839.0 (485.7, 4364.0)	< 0.001
NT-proBNP peak, ng/ml	910.0 (386.0, 2087.5)	2272.0 (882.0, 5778.5)	< 0.001
FIB, g/L	3.01 (2.43, 3.96)	3.29 (2.63, 4.24)	< 0.001
**Angiogram (*n* = 8,013)**			
**Number of lesioned vessels**			
One, n (%)	1082 (46.0)	1771 (31.9)	< 0.001
Two, n (%)	836 (35.3)	2042 (36.2)	0.427
Three, n (%)	453 (19.2)	1829 (32.9)	< 0.001
Left main lesion, n (%)	84 (3.6)	422 (7.6)	< 0.001

CHD, Coronary heart disease; CKD, Chronic kidney disease; PAD, Peripheral arterial disease; HR, Heart rate; MAP, Mean arterial pressure; LVEF, Left ventricular ejection fraction; WBC, White blood cell count; Hb, Hemoglobin; PLT, Platelet count; GLU, Blood glucose; Cr, Creatinine; UA, Uric acid; TG, Triglyceride; TC, Total cholesterol; LDL-C, Low-density lipoprotein cholesterol; HDL, High-density lipoprotein cholesterol; K^+^, Serum potassium level; cTnT, Cardiac troponin T; NT-proBNP, N terminal pro B type natriuretic peptide; FIB, Fibrin. Overweight defined as BMI 25∼30 kg/m^2^; obesity defined as BMI > 30 kg/m^2^.

As for the pathogeny of AMI, coronary atherosclerosis accounted for the largest proportion (95.4%) and was also a more significant cause of infarction in the non-premature AMI group compared to the premature AMI group (90.8 vs. 97.4%, *P* < 0.001) (Supplementary Table 3). Non-atherosclerotic factors such as coronary artery aneurysm (1.5 vs. 0.2%, *P* < 0.001), spontaneous coronary artery dissection (SCAD, 1.6 vs. 0.1%, *P* < 0.001), coronary artery spasm (0.7 vs. 0.2%, *P* < 0.001), myocardial bridge (1.1 vs. 0.1%, *P* < 0.001), coronary embolism (1.2 vs. 0.5%, *P* = 0.002) and coronaritis (0.1 vs. 0%, *P* = 0.026) were more common in the premature AMI group. Additionally, patients with premature AMI had more ST-segment elevation on ECG (65.8 vs. 53.7%, *P* < 0.001).

### Clinical features

Compared to the non-premature AMI group, patients with premature AMI tend to have typical chest pain as an onset symptom (51.6 vs. 45.2%, *P* < 0.001). Patients with premature AMI are most commonly seen with anterior wall infarction suggested by electrocardiography (36.4%). As for heart function, the non-premature AMI group tended to have a poorer heart function, as evidenced by the higher number of patients with low left ventricular ejection fraction (LVEF) (LVEF < 50%) (31.4 vs. 39.5%, *P* < 0.001) and the Killip classification of II-IV (30.2 vs. 54.5%, *P* < 0.001). Simultaneously, higher median NT-proBNP peak levels (910.0 vs. 2272.0 ng/ml, *P* < 0.001) (Table 1).

As for the laboratory tests, the premature AMI group had higher white blood cell count (WBC, 10.2 × 10^9^ vs. 8.9 × 10^9^/L, *P* < 0.001), Hb (145 vs. 130 g/L, *P* < 0.001), and platelet (187 × 10^9^ vs. 162 × 10^9^/L, *P* < 0.001) levels. Correspondingly, the non-premature AMI group had higher blood glucose (7.16 vs. 7.51 mmol/L, *P* = 0.003), creatinine (77 vs. 86 μmol/L, *P* < 0.001), cTnT (1607.5 vs. 1839.0 ng/L, *P* < 0.001), and fibrinogen (3.01 vs. 3.29 g/L, *P* < 0.001) levels. In patients who underwent coronary angiography (*n* = 8,013), the number of left main stem lesions (3.6 vs. 7.6%, *P* < 0.001) was greater in the non-premature AMI group and single branch lesion (46.0 vs. 31.9%, *P* < 0.001) were more common in patients with early-onset AMI (Table 1).

### Outcomes

In our study, the primary outcome is all-cause death at a post-discharge follow-up. A total of 8,527 patients were discharged. The median follow-up time was 4.6 (IQR 2.8–6.9) years, with a missed follow-up rate of 8.23%. The number of overall all-cause death was 1356 (15.9%). Mortality in the premature AMI group was significantly lower than in the non-premature AMI group (5.6 vs. 20.1%, *P* < 0.001).

**FIGURE 2:**
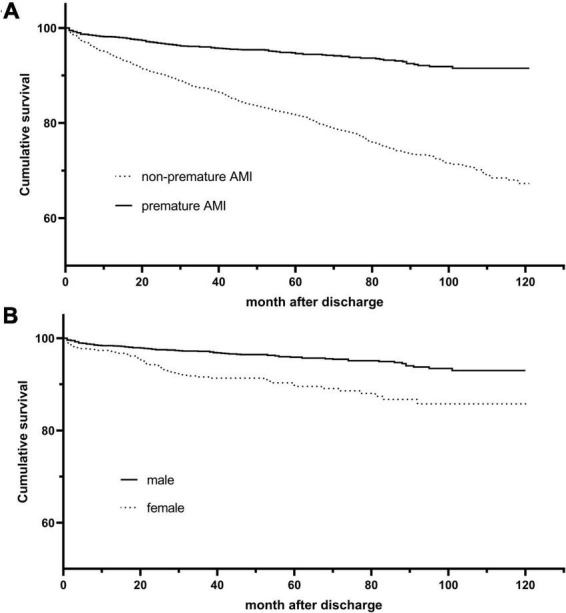
Kaplan-Meier survival curves for overall and premature acute myocardial infarction (AMI) patients. **(A)** Kaplan–Meier curves for all-cause death stratified by age group (log-rank, *P* < 0.001). **(B)** Kaplan–Meier curves for all-cause death stratified by gender (log-rank, *P* < 0.001).

**FIGURE 3:**
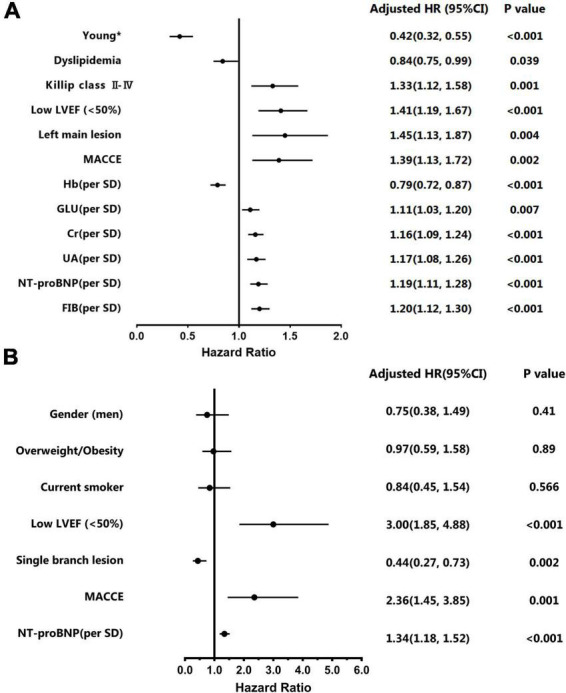
Multivariate Cox regression models for overall and premature acute myocardial infarction (AMI) patients. **(A)** Adjusted Hazard ratios and 95% confidence intervals of multivariate for overall patients. **(B)** Adjusted Hazard ratios and 95% confidence intervals of multivariate for young patients. *The group aged < 55 years in men and < 65 years in women.

According to the Kaplan-Meier survival curve (Figure 2A), the cumulative survival rate of patients in the premature AMI group was significantly better than that of patients in the non-premature group at the 4.6-year follow-up (HR = 0.27, 95% CI 0.22–0.32, p_log–rank_ < 0.001). Also, Killip classification II-IV (Adjusted HR = 1.33, 95% CI 1.12–1.58, *p* = 0.001), reduced LVEF (Adjusted HR = 1.41, 95% CI 1.19–1.67, *p* < 0.001), left main trunk lesion (Adjusted HR = 1.45, 95% CI 1.13–1.87, *p* = 0.004), incidence of in-hospital major adverse cardiac and cerebrovascular events (MACCEs) (Adjusted HR = 1.39, 95% CI 1.13–1.72, *p* = 0.002), high blood glucose (Adjusted HR = 1.11, 95% CI 1.03–1.20, *p* = 0.007),creatinine (Adjusted HR = 1.16, 95% CI 1.09–1.24, *p* < 0.001), uric acid (Adjusted HR = 1.17, 95% CI 1.08–1.26, *p* < 0.001),NT-proBNP (Adjusted HR = 1.19, 95% CI 1.11–1.28, *p* < 0.001) and fibrinogen (Adjusted HR = 1.20, 95% CI 1.12–1.30, *p* < 0.001) levels were risk factors for poor out-of-hospital prognosis (Figure 3A and Supplementary Table 4).

Secondary outcomes included all-cause death, cardiac death, and MACCEs in the hospital. In our study, the in-hospital mortality was 4.6% (*n* = 415), and the incidence of combined MACCEs in the hospital was 15.9% (*n* = 1,420). In general terms, the in-hospital prognosis including all-cause death (1.9 vs. 5.7%, *P* < 0.001), cardiac death (1.7 vs. 5.1%, *P* < 0.001), and MACCEs (12.7 vs. 17.1%, *P* < 0.001) were worse in the non-premature AMI group (Table 2).

**TABLE 2:** Comparison of clinical outcomes between the two groups.

	Premature AMI	Non-premature AMI	*P*-value
**Primary endpoint**			
All-cause death (out-of-hospital)	138 (5.6)	1218 (20.1)	*P* < 0.001
**Second endpoints**			
All-cause death (in hospital)	48 (1.9)	367 (5.7)	*P* < 0.001
Cardiovascular death (in hospital)	43 (1.7)	331 (5.1)	*P* < 0.001
MACCEs (in hospital)	318 (12.7)	1102 (17.1)	*P* < 0.001
Cardiogenic shock	124 (4.9)	490 (7.6)	*P* < 0.001
Malignant arrhythmia	171 (6.8)	713 (11.1)	*P* < 0.001
Mechanical complications	53 (2.1)	175 (2.7)	0.098
Non-fatal stroke	17 (0.7)	29 (0.5)	0.180
Non-fatal recurrent MI	2 (0.1)	4 (0.1)	0.776

MACCEs, Major adverse cardiovascular and cerebrovascular events; MI, Myocardial infarction.

### Gender subgroup analysis

For a gender subgroup analysis of the premature AMI group, the median age of male patients was 48 (43, 51) years and 59 (51.5, 62) years for female patients. The proportion of male patients who smoke was significantly higher than that of female patients (81.3 vs. 3.9%, *P* < 0.001). Risk factors such as hypertension (28.3 vs. 47.8%, *P* < 0.001), diabetes (13.0 vs. 28.7%, *P* < 0.001), and a history of AMI (6.2 vs. 11.6%, *P* < 0.001) were more common in female patients, while dyslipidemia (78.5 vs. 63.6%, *P* < 0.001), overweight or obesity (71.6 vs. 38.0%, *P* < 0.001), and a family history of CHD (7.6 vs. 4.7%, *P* = 0.022) were more common in male patients. As shown in Supplementary Table 3, compared to female patients with premature AMI, atherosclerosis is a more common pathogeny in male patients (92.2 vs. 85.0%, *P* < 0.001). In the meantime, non- atherosclerosis cause was more prevalent in young women.

As for heart function, women had a higher proportion of Killip classification II-IV (26.5 vs. 44.7%, *P* < 0.001) and NT-proBNP peak level (796.0 vs. 1567.5 ng/ml, *P* < 0.001), while there were no gender differences in LVEF. In terms of outcomes, female patients had higher in-hospital (1.5 vs. 3.3%, *P* = 0.008) and out-of-hospital (4.3 vs. 10.9%, *P* < 0.001) all-cause mortality than male patients (Table 3).

**TABLE 3:** Gender grouping analysis of premature acute myocardial infarction (AMI) patients.

	Men (*n* = 2,005)	Women (*n* = 508)	*P*-value
Age, years	48 (43, 51)	59 (51.5, 62)	< 0.001
Current smoker, n (%)	1631 (81.3)	20 (3.9)	< 0.001
Hypertension, n (%)	568 (28.3)	243 (47.8)	< 0.001
Diabetes, n (%)	261 (13.0)	146 (28.7)	< 0.001
Dyslipidemia, n (%)	1574 (78.5)	323 (63.6)	< 0.001
Overweight/Obesity, n (%)	1435 (71.6)	193 (38.0)	< 0.001
Family history of CHD, n (%)	153 (7.6)	24 (4.7)	0.022
ST-segment elevation, n (%)	1343 (67.0)	310 (61.0)	0.011
Left main lesion, n (%)	62 (3.3)	22 (4.8)	0.107
LVEF < 50%, n (%)	527 (31.3)	140 (31.9)	0.822
Killip class II–IV, n (%)	532 (26.5)	227 (44.7)	< 0.001
NT-proBNP peak, ng/ml	796.0 (346.0, 1755.0)	1567.5 (654.0, 4055.0)	< 0.001
**Out-of-hospital outcomes**			
All-cause death	86 (4.3)	52 (10.9)	< 0.001
**In-hospital outcomes**			
All-cause death	31 (1.5)	17 (3.3)	0.008
cardiovascular death	30 (1.5)	13 (2.6)	0.099
MACCEs	242 (12.1)	76 (15.0)	0.080
Cardiogenic shock	89 (4.4)	35 (6.9)	0.023

CHD, Coronary heart disease; LVEF, Left ventricular ejection fraction; NT-proBNP, N terminal pro B type natriuretic peptide; MACCEs, Major adverse cardiovascular and cerebrovascular events.

The Kaplan-Meier survival curve shows that the cumulative survival rate is better for male patients than for female patients (HR 0.41, 95% CI 0.29–0.57, P_log–rank_ < 0.001) (Figure 2B). Baseline variables considered clinically relevant or showed a univariate relationship with outcome were entered into the multivariate Cox proportional-hazards regression model (Figure 3B and Supplementary Table 5). Variables for inclusion were carefully chosen, given the number of events available, to ensure parsimony of the final model. As the multivariate Cox model suggested, low LVEF (Adjusted HR 3.00, 95% CI 1.85–4.88, *P* < 0.001) on echocardiography during hospitalization, elevated NT-proBNP peak level (Adjusted HR 1.34, 95% CI 1.18–1.52, *P* < 0.001) and the occurrence of in-hospital MACCEs (Adjusted HR 2.36, 95% CI 1.45–3.85, *P* = 0.001) were predictors of poor prognosis in young patients. Correspondingly, patients with single branch lesions (Adjusted HR 0.44, 95% CI 0.27–0.73, *P* = 0.002) had a better prognosis.

## Discussion

This retrospective study has several findings. Firstly, the number of patients with premature AMI increased every year. Secondly, lifestyle-related risk factors such as smoking, dyslipidemia, overweight, and obesity, together with a family history of CHD, are independent risk factors for premature AMI. Thirdly, patients with premature AMI have better coronary angiographic phenotype and post-infarction cardiac function than patients with non-premature AMI. Finally, the cumulative survival rate of patients in the premature AMI group was significantly better than that of patients in the non-premature group at the 4.6-year follow-up. Low LVEF, elevated NT-proBNP peak level, and the occurrence of in-hospital MACCEs were predictors of poor prognosis in young patients.

Previous studies have shown that the proportion of men with AMI in the young population was higher than that of women (7, 17). The lower incidence of AMI in young women is thought to be due to the protective effect of circulating estrogen on the vascular endothelium (18). Arora’s study had shown that the annual proportion of AMI admissions attributable to young patients steadily increased from 1995 to 2013, with the most significant increase observed in women (5), which is partially similar to the present study. The increase observed in our study was significant in the premature AMI population and young males. Even though this study did not observe a rise in the proportion of female patients as in Arora’s study, there was no significant decline, indicating that young female patients should not be overlooked. Also, like Arora’s study, this study observed a more significant burden of hypertension and diabetes comorbidity in women than in men with premature AMI.

Risk factors in patients with premature AMI are mostly modifiable compared to older patients (8, 17, 19). In our study, the risk factors for morbidity in young patients mainly were smoking, hyperlipidemia, overweight, or obesity, which are risk factors associated with poor lifestyles. Similar to the previous study (17), we also observed that 97.8% of young patients had at least one risk factor at the time of development of AMI. All these results suggest the importance of changing poor lifestyles for the primary prevention of AMI in the young population.

Regarding lipid metabolism, patients in the premature AMI group were worse than those in the non-premature AMI group, with higher TG, TC, and LDL-C levels and lower HDL-C levels. The characteristics of young patients in terms of lipid metabolism are primarily due to factors such as poor lifestyle and dietary habits. At the same time, some patients may also have lipid metabolism disorders due to unrecognized and uncorrected familial hypercholesterolemia (FH), which predisposes them to earlier coronary heart disease (20, 21). This emphasizes that early screening, diagnosis, effective pharmacological interventions for FH, and lifestyle optimization such as exercise and diet to maintain normal lipid levels are meaningful primary and secondary prevention strategies for younger patients (22, 23).

It was clear that the cumulative survival rate was higher in patients with premature AMI than those with non-premature AMI. Relevant factors may be that younger patients tend to have single-branch lesions and fewer multiple-branch or left main lesions. Cardiac function is less affected by infarction in younger patients (6, 7). Also, there were gender differences in cumulative survival rates in the subgroup analysis of early-onset patients. Young female patients had higher out-of-hospital all - cause mortality than male patients. Similar results have been found in previous studies. Possible influencing factors may be that female patients often have a combination of Diabetes Mellitus (DM), hypertension, and cardiogenic shock (5, 18, 24). Another factor may be that female MI patients often present with atypical chest pain, leading to a delay in hospitalization and thus affecting the timeliness of reperfusion (7, 18, 25). In our study, there was no difference in adjusted out-of-hospital mortality between men and women, suggesting that gender differences were influenced by confounding factors. The same result was reached in the Vienna STEMI registry study (26), while the opposite conclusion was given in the SWEDEHEART study (27).

In our study, as the multivariate Cox model suggested, low LVEF, elevated NT-proBNP peak level, and the occurrence of in-hospital MACCEs were predictors of poor prognosis in young patients. Correspondingly, patients with single branch lesions had a better prognosis. This further emphasizes the need for enhanced out-of-hospital management of young patients with poor cardiac function or developing MACCEs during hospitalization. Notably, dyslipidemia did not increase out-of-hospital all-cause mortality in the study, both in the overall and premature AMI group. Gao’s study, which used recurrent MI as the end event, reached similar conclusions (28). In Winter’s study, non-HDL and remnant cholesterol are strongly associated with unfavorable outcomes in patients with premature myocardial infarction. At the same time, LDL and HDL revealed no significant impact on cardiovascular outcome (29). That may be because a previous study has shown that patients with premature coronary artery disease had a specific high-risk lipid phenotype with a predominance of elevated triglyceride-rich lipoproteins (30). It seems that non-HDL or remnant cholesterol levels are much stronger associated with premature myocardial infarction than HDL or LDL (31, 32). At the same time, the antioxidant function of HDL was compromised in young patients during the acute phase of AMI and the chronic stable phase 1 year after the event, which perhaps also influenced the positive prognostic effect of HDL (33).

Smokers have lower mortality after AMI than non-smokers. The theory is known as the smoker’s paradox (34). Redfors’ study came to the opposite conclusion that, after adjustment for age and other risk factors, smokers had a similar 1-year risk of death and higher risks of death or HF hospitalization as well as reinfarction (35). This may be because smokers were, on average, a decade younger than non-smokers (35, 36). The good prognosis associated with a young age is the main reason for arriving at the smoker’s paradox. After adjustment with multivariate Cox regression, the present study found that smoking was not a protective factor for out-of-hospital mortality in the overall patients and the premature AMI group and became statistically insignificant. This suggests that the beneficial prognostic effects of smoking are due to confounding factors.

Some other risk factors for premature coronary artery disease have been identified in other research. Felice and co-workers confirmed that Lp(a) level is a risk factor (37). More research on premature coronary artery disease should be carried out.

This study had some potential limitations. Some issues inherent to this type of study are related to retrospective data collection and analysis of the data. The sample included in this study spanned a considerable period. Changes in the means of treatment also impacted patient prognosis, which affected the study results. In young patients with AMI, in addition to the traditional risk factors, triggers such as high mental stress and exertion also play a role in the onset of AMI were not collected. Only out-of-hospital all-cause death was collected as a long-term prognosis in this study. It would be more relevant to analyze the factors influencing out-of-hospital cardiac death, cardiovascular events, and recurrent myocardial infarction in younger patients. The study of risk factors and clinical features in this work can help clinical practitioners with their clinical decisions by (1) improving prognostic assessment; (2) intensifying the control of risk factors and clinical features.

## Conclusion

The number of patients with premature AMI has been increasing in recent years. AMI in young patients is associated with an unhealthy lifestyle: smoking, dyslipidemia, and obesity. This highlights the importance of lifestyle for primary and secondary prevention of AMI in young populations (38). Low LVEF, elevated NT-proBNP peak level, and the occurrence of in-hospital MACCEs were predictors of poor prognosis in premature AMI patients.

## Data availability statement

The original contributions presented in this study are included in the article/Supplementary material, further inquiries can be directed to the corresponding authors.

## Ethics statement

The studies involving human participants were reviewed and approved by the Ethics Committee of the West China Hospital of Sichuan University (2012-243). Written informed consent was obtained from the individual(s) for the publication of any potentially identifiable images or data included in this article.

## Author contributions

QL and R-JS contributed to the writing, revision, and statistical analysis of the manuscript. Y-MZ, Y-HC, B-SY, and Y-KZ contributed to the data collection. B-TH and MC had the idea of the manuscript. All authors contributed to the article and approved the submitted version.
