# Supplementary material for: Risk factors, clinical features, and outcomes of premature acute myocardial infarction
Source: Front Cardiovasc Med. 2022 Nov 30;9:1012095. doi: 10.3389/fcvm.2022.1012095 (PMC9747765; doi:10.3389/fcvm.2022.1012095)
Supplement: Supplementary file 1 [file Data_Sheet_1.docx]

SUPPLEMENTARY MATERIALS

**Supplementary Table 1** Joint point regression of the composition of the premature AMI group, 2011-2019

|  | APC | Lower CI | Upper CI | P for trends |
| --- | --- | --- | --- | --- |
| Overall | 2.85 | 0.90 | 4.90 | 0.011 |
| Female | -1.70 | -4.30 | 1.00 | 0.176 |
| Male | 4.10 | 1.90 | 6.40 | 0.003 |

APC: Annual percentage change; CI: Confidence interval.

**Supplementary Table 2** Comparison of the number of risk factors between the two groups

| Number of risk factors | Premature AMI | Non-premature AMI | P-value |
| --- | --- | --- | --- |
| 0, n (%) | 55(2.2) | 506(7.9) | <0.001 |
| ≥1, n (%) | 2458 (97.8) | 5923(92.1) | <0.001 |
| ≥2, n (%) | 2156 (85.8) | 4141(64.4) | <0.001 |
| ≥3, n (%) | 1391 (55.4) | 1834(28.5) | <0.001 |
| ≥4, n (%) | 484 (19.3) | 427(6.64) | <0.001 |
| ≥5, n (%) | 75 (2.98) | 47(0.73) | <0.001 |
| 6, n (%) | 7(0.3) | 1(0.01) | 0.001 |

Risk factors include: current smoker; Hypertension; Diabetes; Dyslipidemia; overweight/obesity; family history of CHD.

**Supplementary Table 3** Pathogeny by age and sex (n=8013)

| Pathogeny | Premature AMI（n=2371） | | | Non-premature AMI | P value |
| --- | --- | --- | --- | --- | --- |
|  | Male(n=1911) | Female(n=460) | P value |  |  |
| Atherosclerosis | 2153（90.8%） | | - | 5493（97.4%） | ＜0.001 |
|  | 1762(92.2%) | 391(85.0%) | ＜0.001 | - | |
| CAA | 35（1.5%） | | - | 9（0.2%） | ＜0.001 |
|  | 30(1.6%) | 5(1.1%) | 0.441 | - | |
| SCAD | 37（1.6%） | | - | 7（0.1%） | ＜0.001 |
|  | 13(0.7%) | 24(5.2%) | ＜0.001 | - | |
| CAS | 17（0.7%） | | - | 11（0.2%） | ＜0.001 |
|  | 14(0.7%) | 3(0.7%) | 1.000 | - | |
| MB | 25（1.1%） | | - | 4（0.1%） | ＜0.001 |
|  | 22(1.2%) | 3(0.7%) | 0.492 |  | |
| Coronaritis | 3（0.1%） | | - | 0 | 0.026 |
|  | 1(0.1%) | 2(0.4%) | 0.098 | - | |
| CE | 28（1.2%） | | - | 30(0.5%) | 0.002 |
|  | 22(1.2%) | 6(1.3%) | 0.785 | - | |
| CMD | 2(0.1%) | | - | 3(0.1%) | 0.636 |
|  | 1(0.1%) | 1(0.2%) | 0.350 | - | |
| Unclassified | 71(3.0%) | | | 85(1.5%) | |

CAA: Coronary Artery Aneurysm; SCAD: Spontaneous coronary artery dissection; CAS: Coronary artery spasm; MB: Myocardial Bridge; CE Coronary Embolism; CMD: Coronary Microvascular Dysfunction.

**Supplementary Table 4** Crude and Adjusted Hazard Ratio of variables on the risk of death of patients

|  | **Crude** **Hazard Ratio** | |  | **Adjusted** **Hazard Ratio** | |
| --- | --- | --- | --- | --- | --- |
|  | **HR (95%CI)** | **P value** |  | **HR (95%CI)** | **P value** |
| Gender (men) | 0.61(0.54, 0.68) | <0.001 |  | 0.89(0.72, 1.09) | 0.267 |
| Young* | 0.27(0.22, 0.32) | <0.001 |  | 0.42(0.32, 0.55) | <0.001 |
| Current smoker | 0.56(0.50, 0.63) | <0.001 |  | 0.86(0.72, 1.04) | 0.113 |
| Overweight/obesity | 0.52(0.46, 0.60) | <0.001 |  | 0.94(0.77, 1.14) | 0.532 |
| Hypertension | 1.37(1.23, 1.53) | <0.001 |  | 0.97(0.82, 1.13) | 0.677 |
| Diabetes | 1.60(1.42, 1.80) | <0.001 |  | 0.99(0.82, 1.21) | 0.933 |
| Dyslipidemia | 0.80(0.72,0.89) | <0.001 |  | 0.84(0.75, 0.99) | 0.039 |
| ST-segment elevation | 0.83(0.74, 0.92) | <0.001 |  | 0.88(0.75, 1.03) | 0.107 |
| Killip class Ⅱ-Ⅳ | 2.86(2.54, 3.20) | <0.001 |  | 1.33(1.12, 1.58) | 0.001 |
| Low LVEF (<50%) | 2.44(2.17, 2.74) | <0.001 |  | 1.41(1.19, 1.67) | <0.001 |
| Left main lesion | 2.28(1.88, 2.77) | <0.001 |  | 1.45(1.13, 1.87) | 0.004 |
| Single branch lesion | 0.50(0.43, 0.58) | <0.001 |  | 0.85(0.71, 1.02) | 0.085 |
| MACCE | 3.03(2.69, 3.43) | <0.001 |  | 1.39(1.13, 1.72) | 0.002 |
| WBC, per SD | 1.20(1.14, 1.25) | <0.001 |  | 1.06(0.98, 1.14) | 0.167 |
| Hb, per SD | 0.59(0.56, 0.61) | <0.001 |  | 0.79(0.72, 0.87) | <0.001 |
| GLU, per SD | 1.26(1.21, 1.31) | <0.001 |  | 1.11(1.03, 1.20) | 0.007 |
| Cr, per SD | 1.26(1.23, 1.29) | <0.001 |  | 1.16(1.09, 1.24) | <0.001 |
| UA, per SD | 1.40(1.34, 1.47) | <0.001 |  | 1.17(1.08, 1.26) | <0.001 |
| LDL-C, per SD | 0.82(0.77, 0.87) | <0.001 |  | 1.08(0.99, 1.18) | 0.091 |
| cTnT, per SD | 1.10(1.04, 1.16) | 0.001 |  | 0.96(0.89, 1.04) | 0.355 |
| NT-proBNP, per SD | 1.63(1.58, 1.68) | <0.001 |  | 1.19(1.11, 1.28) | <0.001 |
| FIB, per SD | 1.37(1.31, 1.44) | <0.001 |  | 1.20(1.12, 1.30) | <0.001 |

HR: Hazard Ratio; LVEF: Left ventricular ejection fraction; WBC: White blood cell count; Hb: Hemoglobin; GLU: Blood glucose; Cr: Creatinine; UA: Uric acid; LDL-C: Low-density lipoprotein cholesterol; cTnT: Cardiac troponin T; NT-proBNP: N terminal pro B type natriuretic peptide; FIB: Fibrin.

Overweight/obesity is defined as BMI≥25 kg/m2.

* the group aged <55 years in men and <65 years in women.

**Supplementary Table 5** Crude and Adjusted Hazard Ratio of variables on the risk of death of premature AMI patients

|  | **Crude Hazard Ratio** | |  | **Adjusted** **Hazard Ratio** | |
| --- | --- | --- | --- | --- | --- |
|  | **HR (95%CI)** | **P value** |  | **HR (95%CI)** | **P value** |
| Gender (men) | 0.41(0.29, 0.57) | <0.001 |  | 0.75(0.38, 1.49) | 0.410 |
| Overweight/Obesity | 0.67(0.47, 0.95) | 0.024 |  | 0.97(0.59, 1.58) | 0.890 |
| Current smoker | 0.53(0.38, 0.75) | <0.001 |  | 0.84(0.45, 1.54) | 0.566 |
| Dyslipidemia | 0.82(0.57, 1.18) | 0.281 |  | — | — |
| Low LVEF (<50%) | 4.94(3.35, 7.29) | <0.001 |  | 3.00(1.85, 4.88) | <0.001 |
| Single branch lesion | 0.35(0.22, 0.55) | <0.001 |  | 0.44(0.27, 0.73) | 0.002 |
| MACCE | 6.23(4.43, 8.77) | <0.001 |  | 2.36(1.45, 3.85) | 0.001 |
| NT-proBNP, per SD | 1.51(1.42, 1.61) | <0.001 |  | 1.34(1.18, 1.52) | <0.001 |

LVEF: Left ventricular ejection fraction; MACCE: Major adverse cardiovascular and cerebrovascular events; NT-proBNP: N terminal pro B type natriuretic peptide.


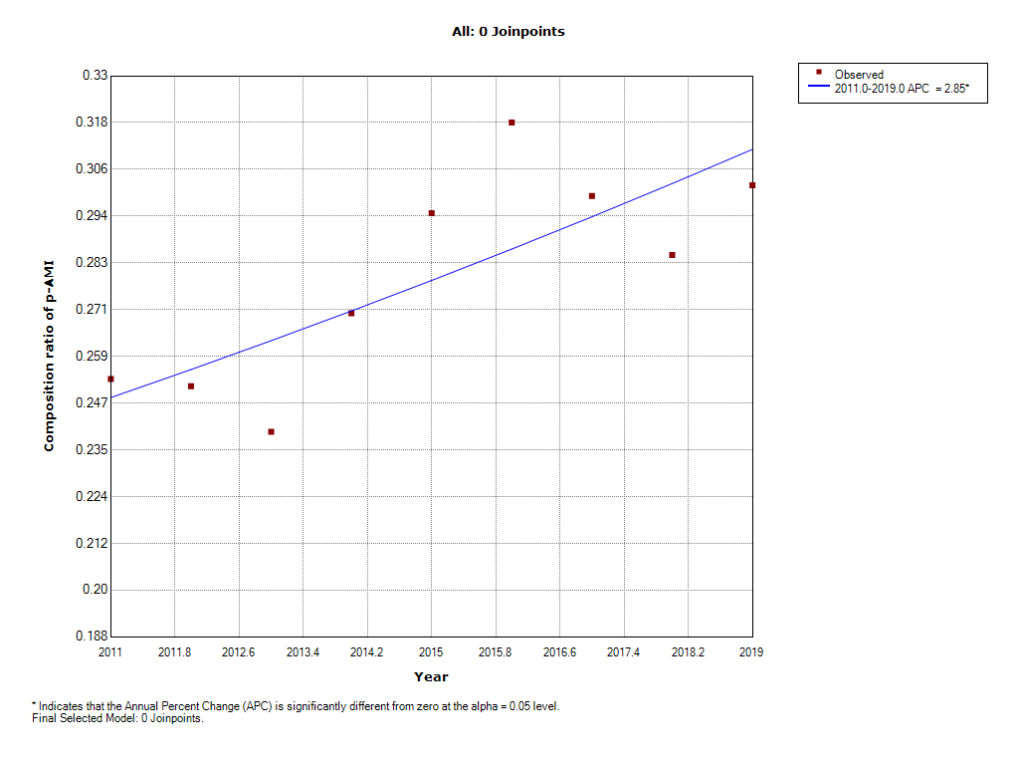

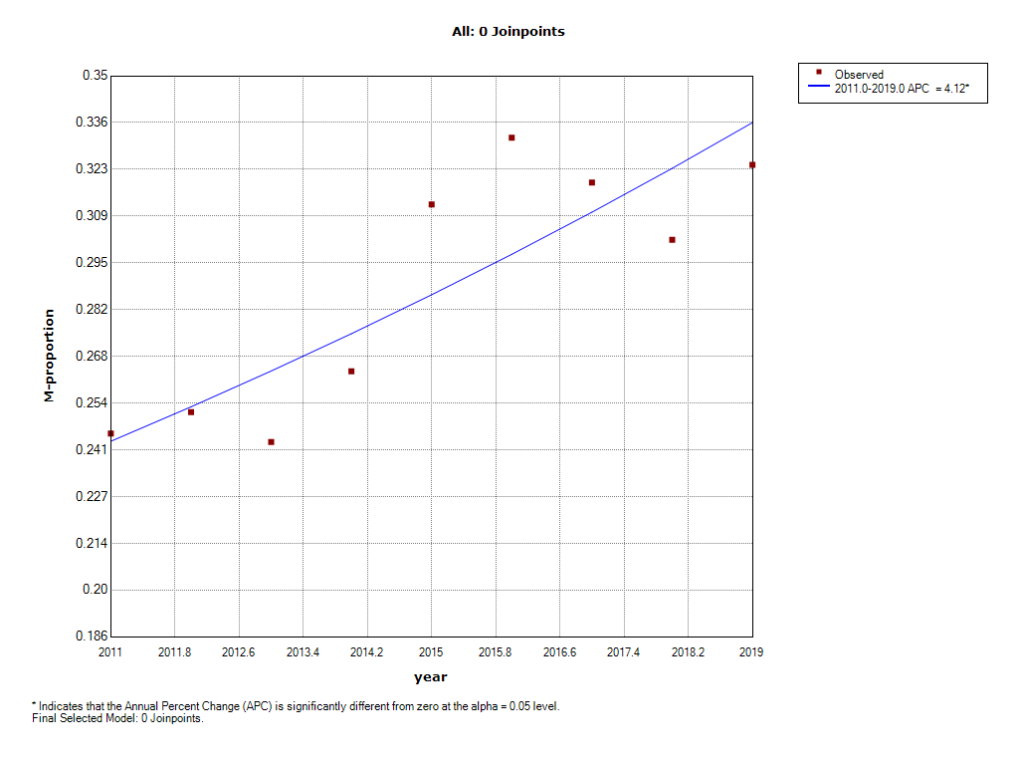


(a)

(b)


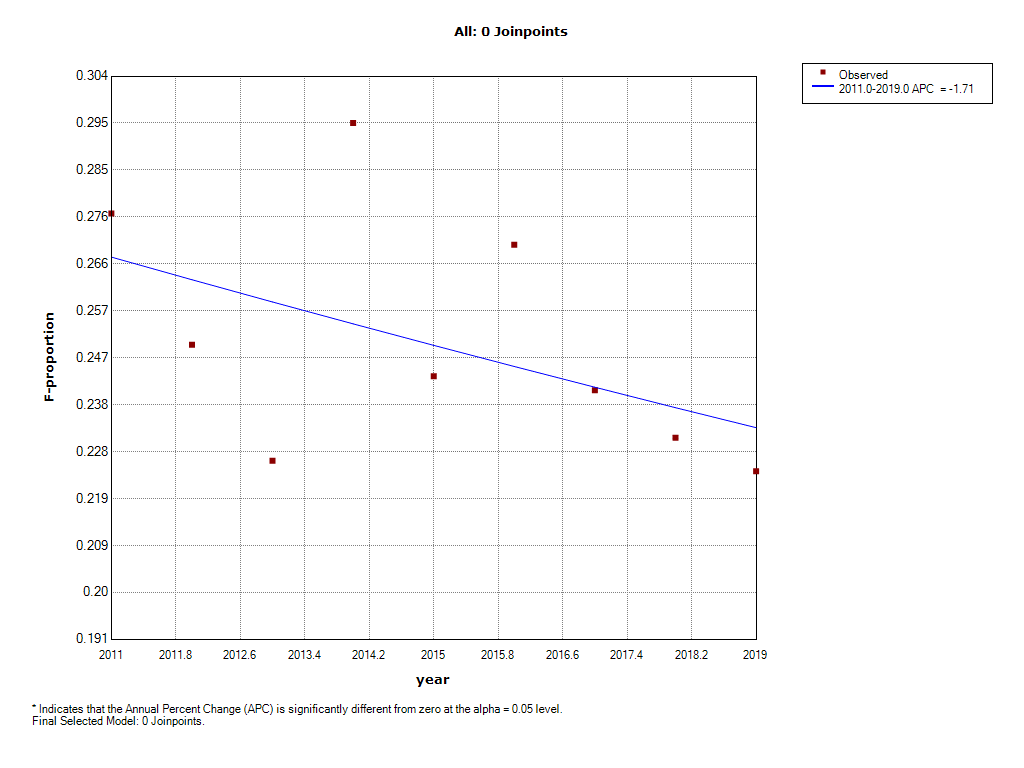


(c)

**Supplementary Figure 1** Joinpoint regression of the year-to-year change in the composition of patients with premature AMI. (a) overall composition of patients with premature AMI; (b) composition of male patients with premature AMI; (c) composition of female patients with premature AMI.

APC: Annual percentage change.
